# NBS-LRR-mediated resistance triggered by aphids: viruses do not adapt; aphids adapt via different mechanisms

**DOI:** 10.1186/s12870-016-0708-5

**Published:** 2016-01-22

**Authors:** Nathalie Boissot, Sophie Thomas, Véronique Chovelon, Hervé Lecoq

**Affiliations:** Institut National de la Recherche Agronomique (INRA), UR1052, Unité de Génétique et Amélioration des Fruits et Légumes, Domaine St Maurice - Allée des chênes, CS 60094, F-84143 Montfavet cedex, France; Institut National de la Recherche Agronomique (INRA), UR452, Unité de Pathologie Végétale, Domaine St Maurice - Allée des chênes, CS 60094, F-84143 Montfavet cedex, France

**Keywords:** *Aphis gossypii*, *Cucumis melo*, *Vat*, Durable resistance, Biotype, Allele, Melon, Transgenic melon

## Abstract

**Background:**

Aphids are serious pest on crops. By probing with their stylets, they interact with the plant, they vector viruses and when they reach the phloem they start a continuous ingestion. Many plant resistances to aphids have been identified, several have been deployed. However, some resistances breaking down have been observed. In the melon, a gene that confers resistance to aphids has been deployed in some melon-producing areas, and aphid colony development on *Vat*-carrying plants has been observed in certain agrosystems. The *Vat* gene is a NBS-LRR gene that confers resistance to the aphid species *Aphis gossypii* and exhibits the unusual characteristic of also conferring resistance to non-persistently transmitted viruses when they are inoculated by the aphid. Thus, we characterized patterns of resistance to aphid and virus using the aphid diversity and we investigated the mechanisms by which aphids and viruses may adapt to the *Vat* gene.

**Results:**

Using a *Vat*-transgenic line built in a susceptible background, we described the *Vat*- spectrum of resistance to aphids, and resistance to viruses triggered by aphids using a set of six *A. gossypii* biotypes. Discrepancies between both resistance phenotypes revealed that aphid adaptation to *Vat*-mediated resistance does not occur only via avirulence factor alterations but also via adaptation to elicited defenses. In experiments conducted with three virus species serially inoculated by aphids from and to *Vat* plants, the viruses did not evolve to circumvent *Vat*-mediated resistance.

We confirmed discrepancies between both resistance phenotypes by testing each aphid biotype with a set of thirteen melon accessions chosen to reflect the natural diversity of the melon. Inheritance studies revealed that patterns of resistance to virus triggered by aphids are controlled by different alleles at the *Vat* locus and at least another locus located at a short genetic distance. Therefore, resistance to viruses triggered by aphids is controlled by a gene cluster.

**Conclusions:**

Under the Flor model, changes in the avirulence gene determine the ability of the pathogen to overcome the resistance conferred by a plant gene. The *Vat* gene belongs to a resistance gene family that fits this pest/pathogen–plant interaction, and we revealed an additional mechanism of aphid adaptation that potentially exists in other interactions between plants and pests or pathogens.

**Electronic supplementary material:**

The online version of this article (doi:10.1186/s12870-016-0708-5) contains supplementary material, which is available to authorized users.

## Background

Among the 4000 known aphid species worldwide, approximately one hundred have exploited the agricultural environment, and their ability to rapidly colonize crops makes aphids serious pests [1]. Once an aphid settles on crops, it simultaneously feeds and reproduces. An aphid pushes its stylets through layers of plant tissue to reach the phloem. The path from the epidermis to the phloem is intercellular, and an aphid salivates while moving along this path, developing a protective sheath against plant defense. When its stylets are tightly inserted in the phloem, an aphid begins removing photoassimilates by continuous fluid ingestion that causes direct damage to the plant. On crops, aphid reproduction is mainly parthenogenetic with telescoping generations what leads to multiple generations on a crop in a single season. Aphid proliferation on plants can cause stunting, severe leaf curling and plant death. Moreover, short probing punctures in cells along the path to the phloem allow the acquisition/transmission of non-persistent viruses [2]. When the stylets reach the phloem, the acquisition/transmission of persistent viruses can occur [3].

The management of aphids infesting crops is clearly challenging. Pesticide sprays are predominantly used to combat aphids. Since the 1980s, many species have developed resistance to insecticides, particularly two cosmopolitan and polyphagous aphids, *Myzus persicae* (Sulz) and *Aphis gossypii* (Glover) [4]. Screening germplasms for plant resistance led to the discovery of accessions in several crop species that displayed resistance to various aphid species. However, the sources of plant resistance to aphids are limited and rare [5], and the relatively high number of resistant accessions discovered in certain species should not mask the fact that resistance to aphid typically arises from a small number of genes with only a few resistance alleles. In practice, plant genes that confer resistance to aphids have been primarily introduced into cultivated varieties of cereals, fruit trees and vegetables, and some resistant varieties have been deployed on a large scale [5]. Evidence from biotypes (*i.e.* clones able to survive, reproduce on and/or cause injury to a cultivated plant that is resistant to other clones of the same species) indicates that some aphid species can adapt to plant resistance genes. The gene *Ag1*, which confers resistance to *Amphorophora agathonica*, was extensively used in raspberry for fifty years before a resistance-breaking biotype appeared [6]. Resistance to *Nasonovia ribisnigri* is conferred by the *Nr* gene in lettuce, and breakdown of this resistance occurred 10 years after the gene’s wide deployment [7]. The Russian wheat aphid *Diuraphis noxia* overcame the resistance gene *Dn4* less than 10 years after the gene had been released in wheat cultivars [8]. Adapted biotypes of *Schizaphis graminum*, were observed prior to the deployment of some resistances in wheat [9]. Our objective was to investigate aphid adaptation to plant resistance in a system in which plant resistance and aphid diversity have been well characterized: *Cucumis melo* and *A. gossypii*.

*Cucumis melo*, originating from Asia [10], is one of the main species within the *Cucurbitaceae* family. *C. melo* is found throughout the world, exhibiting considerable genetic diversity in cultivated and wild genotypes [11]. Melon crops are only colonized by *A. gossypii*, a cosmopolitan aphid. To date, fewer than twenty multilocus genotypes (MLGs), as revealed by 8 SSR markers, have been observed developing colonies on melon plants [12–15]. Resistant melon accessions have been largely described since the 1970s; they originated from East and Far East Asia, Europe, Africa, America [16]. Early open-field studies revealed that melon resistance to the US Southeastern biotype of aphids was ineffective against the Southwestern biotype [17] and *vice versa*. [18]. In the same manner, low resistance levels to *A. gossypii* from Spain were observed in laboratory biotests in melon accessions that exhibited a high level of resistance to French *A. gossypii* [19]. Therefore adapted clones of *A. gossypii* were already observed, prior to the deployment of resistance in melon crops in some regions. Recently, Thomas et al. [20] demonstrated that *A. gossypii* biotypes can be related to MLGs. Clones sharing the same MLG exhibit a similar acceptance on a set of melon accessions (low acceptance as plant resistance phenotype). Nevertheless, the fitness of the clones sharing the same MLG exhibited some variation. *A. gossypii* is an efficient vector of viruses transmitted in a non-persistent manner such as *Cucumber mosaic virus* (CMV), *Zucchini yellow mosaic virus* (ZYMV), *Watermelon mosaic virus* (WMV) and *Papaya ringspot virus* (PRSV) and an efficient vector of the *Cucurbit aphid-borne yellows virus* (CABYV) transmitted in a persistent manner.

In 1987, a melon cultivar in the French catalog, Margot, was declared resistant to aphids for the first time. This resistance has been characterized using two *A. gossypii* clones, NM1 and C9. It is controlled by a major gene, *Vat*, and several quantitative trait loci that have been localized in the melon genome [21]. The *Vat* gene encodes a coiled-coil (CC)-nucleotide-binding site (NBS)-leucine-rich repeat (LRR) protein [22]. The resistance genes belonging to this family are widely assumed to be involved in the specific recognition of pathogen and pest effectors and the activation of plant defense responses [23]. Recently, the efficacy of this resistance was jeopardized in Southeastern France and was overcome in the Lesser Antilles (Thomas S, Vanlerberghe-Masutti F, Mistral P, Loiseau A, Boissot N: Insight into the durability of aphid resistance from the demo-genetic study of *Aphis gossypii* populations in melon crops. Submitted.).

Altogether, this raises two questions: (1) How broad is the resistance conferred by the *Vat* gene in the face of *A. gossypii* diversity? (2) Are broader forms of resistance available other than *Vat*-mediated resistance among genetically diverse melon? Using a set of aphid clones, we revealed a limited spectrum of *Vat* resistance, and we identified melon accessions exhibiting larger spectra controlled by at least another locus linked to the *Vat* gene.

Moreover, melon plants harboring the resistance *Vat* gene are susceptible to viruses when inoculated mechanically or using other aphid species as vectors [24], but these *Vat* plants become resistant to non-persistent viruses when inoculated by the NM1 and C9 *A. gossypii* biotypes [20, 24]. In other words, when transmitting non-persistent viruses, NM1 and C9 biotypes trigger resistance to these viruses [22]. Thus, two additional questions are raised: (1) Do all aphid biotypes, regardless of their ability to circumvent *Vat*-mediated resistance, trigger resistance to viruses? (2) Are viruses able to adapt to *Vat*-mediated resistance? We showed that clone ability to colonize *Vat*-plants was not a predictor of a lack of ability to trigger the resistance to virus. We also showed that viruses were not able to adapt to *Vat*-mediated resistance.

## Results

### Patterns of resistance observed in a *Vat*-transgenic line and 13 melon accessions with nine *A. gossypii* clones (Data set in Additional file 1)

We observed 125 plant-aphid-virus interactions, 13 melon lines interacting with 9 aphid clones and two transgenic lines interacting with either 6 or 2 clones. These interactions were characterized for three traits, ‘Plant response to CMV’ triggered by aphids, ‘Acceptance’ and ‘Colonization’ by aphids. The biotests were conducted from 2004 to 2015. Margot and Védrantais were included in all tests and used as references to standardize the results obtained over years.

#### Scoring of the two reference lines Védrantais and Margot

**Fig. 1:**
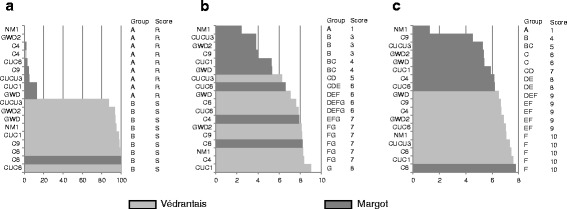
Scoring for three traits of 2 references lines x 9 aphid clones combinations. **a** Percentage of melon plants with CMV symptoms after inoculation by nine clones of *A. gossypii*. Group: significant difference (*p* = 0.05) based on pairwise comparison by χ^2^ statistics with Bonferroni correction (p_cor_ = 0.0006). Score: score for each combination. **b** Acceptance = number of aphids on the plant 72 h after infestation by 10 aphids. Group: significant difference (*p* = 0.05) after a non-parametric test (Steel-Dwass-Critchlow-Fligner procedure) with Bonferroni correction. Score: score for each combination. **c**. Ability to colonize observed 7 days after infestation by 10 aphids. Group: significant difference (*p* = 0.05) after a non-parametric test (Steel-Dwass-Critchlow-Fligner procedure) with Bonferroni correction. Score: score for each combination

The percentage of plants exhibiting CMV symptoms after inoculation by an aphid clone was a quantitative trait (Fig. 1a). Then Védrantais and Margot/ aphid clone interactions were scored S (for susceptible) or R (for resistant). Védrantais was susceptible to CMV inoculated by all clones with the exception of C4, and Margot was resistant to CMV inoculated by all clones with the exception of C6.

‘Acceptance’ was estimated as the number of adult aphids remaining on a plantlet three days after infestation by 10 adults and ranged from 2.4 to 9.0 for the reference lines (Fig. 1b). This trait was quantitative and ‘Acceptance’ scores were determined as follows. Because the lowest ‘Acceptance’ was observed for NM1 on Margot, a score of 1 was given for this interaction. A score of +2, *i.e.* 3, was given to the interaction exhibiting the closest significantly different ‘Acceptance’ from the NM1/Margot interaction, *i.e.* the CUCU3/Margot interaction. A score of 5 was given to the next interaction exhibiting the closest significantly different ‘Acceptance’ from the CUCU3/Margot interaction, *i.e.* CUCU3/Védrantais, and so on. Intermediate scores were given when the differences were not significant. ‘Acceptance’ scores ranged from 1 to 8.

‘Colonization’ was calculated from the number of nymphs and adults on a plantlet 7 days after infestation. This trait also was quantitative ranging from 1.3 to 7.8 (Fig. 1c). A score of 1 was given for the lowest ‘Colonization’ observed (NM1/Margot). The next result observed (C9/Margot) that was significant and largely greater than the first result was given a score of +3, *i.e.* 4. The same procedure outlined for ‘Acceptance’ was followed (+2 was given when the difference was significant). ‘Colonization’ scores ranged from 1 to 10.

These scores were used as references when analyzing the following interactions.

#### Vat resistance spectrum defined on a Vat transgenic line

The *Vat* resistance spectrum to 6 clones of *A. gossypii* was established using the *Vat* transgenic line TR3 based on the three parameters described above. Scores for the three parameters were given to each combination (transgenic line/aphid clone) in comparison to the scores given for each combination ‘reference line/ aphid clone’.

The TR3 line was resistant to CMV when inoculated by C9, GWD2 and NM1 clones proving that the gene was efficient where it was inserted in Védrantais (Table 1). TR3 was susceptible to CMV when inoculated by C6, CUC1 and GWD clones. To confirm that susceptibility to CMV in TR3 was not due to an insertion effect, CMV was inoculated by NM1 and GWD to another transgenic line, TR4. In accordance with results obtained on TR3, TR4 was resistant to CMV when inoculated by NM1 and susceptible to CMV when inoculated by GWD.

**Table 1:** Scores for CMV for 125 melon/aphid interactions

	Plant response to CMV when inoculated by aphids								
	C6	C9	CUC1	CUC6	CUCU3	GWD	GWD2	NM1	C4
^a^TR3	S	R	IS	nt^b^	nt	S	R	R	nt
^a^TR4	nt	nt	nt	nt	nt	S	nt	R	nt
^a^PI 482398	R	R	R	R	R	R	R	R	R
^a^Margot	S	R	R	R	R	R	R	R	R
^a^AM51	S	R	R	R	R	R	R	R	R
^a^PI 161375	S	R	R	R	R	R	R	R	R
^a^San Ildefonso	S	R	R	R	R	ns	R	R	R
Smith Perfect	R	R	R	R	R	R	R	S	R
Canton	I	R	R	R	R	R	R	S	R
HSD2455	R	R	R	ns^c^	R	R	S	S	R
Anso 77	S	S	S	S	R	R	R	R	R
PI 224770	S	S	S	S	S	S	R	R	R
90625	S	S	S	S	S	S	S	R	R
PI 164723	S	S	S	S	S	S	S	R	R
Védrantais	S	S	S	S	S	S	S	S	R

^a^lines or accessions amplifying the Z1431 marker designed from the *Vat* gene [21]

^b^nt untested, ^c^ns not assigned to a class because the differences with controls were not significant in the biotest

Plant response to CMV when inoculated by 9 clones of *A. gossypii* (*R* Resistant, *I* Intermediate and *S* Susceptible) on two *Vat*-transgenic melon lines, TR3 and TR4, and 13 melon accessions. Blank lines separate the CMV resistance patterns

The line TR3 was poorly accepted and poorly or moderately colonized by the C9 and NM1 clones (Table 2) as expected from the resistant phenotype observed when both clones inoculated CMV into TR3 line (Table 1). The TR3 line was moderately accepted and fully colonized by the C6, CUC1, GWD and GWD2 clones (Table 2), and this result is consistent with TR3 susceptibility to CMV inoculated by these aphid clones with the exception of GWD2 which does trigger resistance to CMV (Table 1). Therefore C6, CUC1, GWD and GWD2 clones were adapted to the resistance mediated by *Vat* gene.

**Table 2:** Scores for *A. gossypii* acceptance and colonization for 123 melon/aphid interactions

	Acceptance										Colonization							
	C6	C9	CUC1	CUC6	CUCU3	GWD	GWD2	NM1	C4	C6	C9	CUC1	CUC6	CUCU3	GWD	GWD2	NM1	C4
^a^TR3	6	3	6	nt^b^	nt	6	5	1	nt	10	4	9	nt	nt	9	7	3	nt
^a^PI 482398	7	1	4	6	1	2	3	1	7	12	4	7	8	2	3	6	1	6
^a^Margot	7	3	4	6	3	4	3	1	7	10	4	7	8	5	6	6	1	8
^a^AM51	8	3	4	5	1	2	3	1	5	10	4	4	8	5	3	6	1	6
^a^PI 161375	6	3	4	3	3	4	3	1	7	11	6	7	8	5	6	6	1	9
^a^San Ildefonso	6	3	4	6	3	4	3	1	7	10	9	7	9	5	6	6	1	9
Smith Perfect	7	3	4	6	3	2	3	7	7	12	4	7	8	5	6	4	10	8
Canton	6	3	4	6	3	5	2	7	7	9	1	7	7	5	6	6	10	10
HSD2455	6	3	4	6	3	4	5	4	7	10	7	7	9	5	6	9	5	10
Anso 77	7	3	4	5	3	4	5	1	6	9	4	4	8	5	6	10	1	9
PI 224770	6	5	8	6	5	4	7	1	7	10	9	7	9	10	9	9	1	9
90625	7	7	8	6	5	5	7	−1	6	11	9	10	8	10	8	9	1	6
PI 164723	7	7	9	6	5	5	8	1	6	11	9	10	9	10	9	9	1	7
Védrantais	6	7	8	6	5	6	7	7	7	10	9	10	9	10	9	9	10	9

^a^lines or accessions amplifying the Z1431 marker designed from the *Vat* gene [21]

^b^nt untested

Acceptance by 9 clones of aphid [−1 to 9] and Ability to colonize plant [1–12] of the 9 clones on a *Vat*-transgenic melon line, TR3, and 13 melon accessions

#### Resistance to aphid and resistance to virus triggered by aphids in natural melon diversity

The spectrum of resistance across natural melon diversity was established using 13 melon accessions infested with nine *A. gossypii* clones. Scores for the three parameters were given to each combination (transgenic line/aphid clone) in comparison to the scores given for each combination ‘reference line/aphid clones’.

We revealed eight patterns of resistance to CMV triggered by aphids across the natural melon diversity (separated by blank lines in Table 1). All melon accessions were resistant to CMV when inoculated by C4, even Védrantais, which is typically considered a susceptible control. To check if C4 was an efficient vector of CMV we observed a set of Cucurbits inoculated by CMV using C4. Several melon lines, zucchini squash and cucumber exhibited symptoms (Additional file 2) proving that C4 was able to transmit CMV. Other aphid clones were able to inoculate CMV to Védrantais, proving their vectoring capacity. Sixty percent of the interactions (melon accession/aphid clone) exhibited a resistant plant response. Surprisingly, PI 161375, the accession used to isolate the *Vat* gene, did not exhibit the same pattern of resistance to CMV as the transgenic line TR3. In the same way, none lines amplifying the marker developed from the *Vat* gene (Z1431) did exhibit the same pattern of resistance to CMV as TR3. PI 482398 was the only accession resistant to CMV when inoculated by all the clones tested.

‘Acceptance’ and ‘Colonization’ were scored from −1 to 9 and 1 to 12, respectively but no accession was poorly accepted and poorly colonized by all aphid clones: *i.e.* no accession exhibited a large resistance spectrum to aphids (Table 2). As a matter of fact, several clones heavily colonized the accessions amplifying the *Vat* gene (C6, CUC6, GWD2 and C4) and then were adapted to the *Vat*-resistance. The least colonized accession was AM51, exhibiting a median score of 5, whereas Margot and PI 161375 exhibited a median colonization score of 6. All other accessions and the TR3 line exhibited higher median colonization scores.

**Fig. 2:**
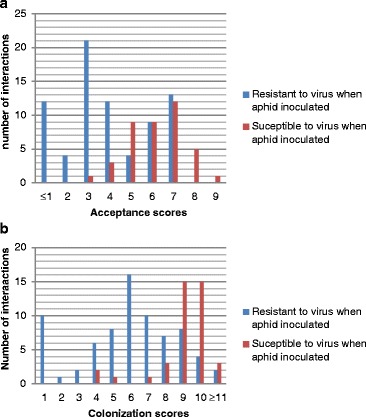
Relationship between resistance to *A. gossypii* and resistance to virus triggered by *A. gossypii*. Distribution of 123 interactions (melon accession/aphid clone) according to aphid **a** Acceptance and **b** Colonization, and interactions exhibiting either a resistant or susceptible phenotype after CMV inoculation by aphids

Of the 123 melon accession /aphid clone interactions we studied, 74 cases of resistance to CMV triggered by aphids were observed. Out of them, 49 cases exhibited low acceptance (Acceptance score ≤4, Fig. 2a) and only 27 exhibited low colonization (Colonization score ≤5, Fig. 2b). For the clone C4, the high colonization of all accessions was discordant with the resistance to CMV triggered by this clone in all accessions. Therefore the resistance to virus triggered by aphids was decoupled from the resistance to aphid: low ‘Acceptance’ was a poor predictor of resistance to virus triggered by aphids (49/74 of convergent results) and low ‘Colonization’ was not a predictor of resistance to virus triggered by aphids and vice-versa (27/74 of convergent results).

### Virus ability to adapt to resistance triggered by aphid

**Fig. 3:**
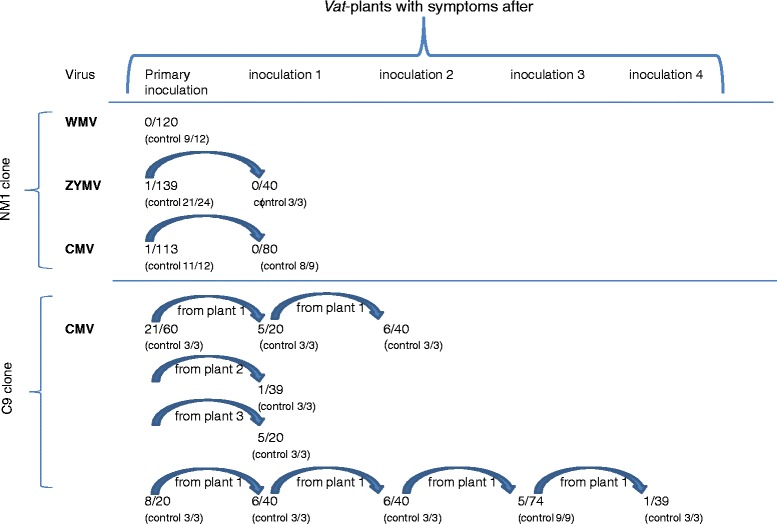
Experimental evolution of WMV, ZYMV and CMV on *Vat* plants. Number of *Vat* plants exhibiting symptoms/number of tested plants after virus inoculation by the NM1 and C9 *A. gossypii* clones*.* For the primary inoculations, aphids acquired viruses from susceptible infected plants. For the following inoculations, aphids acquired viruses from previously infected *Vat* plants. (Control: number of Védrantais plants exhibiting symptoms/number tested)

It is possible that viruses can overcome *Vat*-mediated resistance, by escaping the defense mechanisms induced by an *A. gossypii* effector, and develop systemic infections after serial transmission events on *Vat* plants. To test this hypothesis, sequential virus transmissions from infected *Vat*-carrying Margot plants to healthy Margot plants were established using the NM1 or C9 aphid clones with three viruses, CMV, ZYMV and WMV. No virus evolved in response to resistance triggered by NM1 or C9 (Fig. 3).

When for the first time viruses were inoculated by NM1 into Margot, no WMV infected plants were obtained from the 120 plants tested, one ZYMV infected plant was obtained from the 139 plants tested, and one CMV infected plant was obtained among 113 plants. After back-inoculation from Margot to Margot from the two infected plants, the percentage of plants infected did not increase regardless of the virus tested (Fig. 3).

More infected plants were obtained by the C9 clone than after inoculation by the NM1 clone, thus allowing for easier back-inoculations. Again, we did not observe an increase of the percentage of infected plants, even after the fourth back-inoculation (Fig. 3).

## Discussion

Among all of the known plant genes that confer resistance to aphids [5], the *Vat* gene is unique in that it also confers resistance to viruses when inoculated by aphids [22]. This resistance is restricted to the *A. gossypii* species [25], but its efficacy against the large diversity of *A. gossypii* remains unknown. In addition, allelic variation at the *Vat* locus inducing phenotypical variation has been only hypothetical until now. Given that resistance to virus triggered by aphids was demonstrated to be qualitative, this trait can be used to describe biotypes of *A. gossypii* species. Accordingly to Smith (2005) [26], biotypes are revealed on a set of cultivars, each possessing a different resistance gene or gene combination that react differentially to a given biotype. Based on the plant response of 13 melon accessions to CMV inoculation by 9 aphid clones, *i.e.* 117 interactions, we recognized six aphid biotypes. The first biotype is represented by C6 and triggered resistance to CMV in 4 accessions. The second biotype consists of C9 and CUC1 (and putatively CUC6) and triggered resistance to CMV in 8 accessions. The third biotype, which consists of CUCU3 and putatively GWD, triggered resistance in the same set of accessions as the previous biotype, as well as Anso 77. The fourth and fifth biotypes are represented by GWD2 and NM1, respectively, and triggered resistance to CMV in 9 accessions (some common and some different). The sixth biotype is represented by C4 and triggered resistance to CMV in 13 accessions.

### Are the different patterns of resistance to CMV controlled by the same locus, namely the *Vat* locus?

We revealed an unexpected partial pattern of resistance of the *Vat* gene. The *Vat* gene, isolated from the PI 161375 accession, was characterized using one clone, NM1-Lab [22]. A recent study at the agrosystem level suggested that the *Vat* gene confers resistance to a large number of *A. gossypii* clones [15]. Regarding PI 161375, eight out of the nine clones assessed triggered the resistance to CMV when used as vectors, suggesting again that a large number of *A. gossypii* clones triggers resistance to CMV in *Vat* plants. To confirm this result, we studied this trait in a *Vat* transgenic line. Among five clones triggering high levels of resistance to CMV in PI 161375, only three triggered a high level of resistance to CMV in the transgenic line. These data indicate that at least an additional locus is involved in resistance to CMV triggered by aphid in PI 161375.

On this basis, we proposed to rename the *Vat* locus *Vat-1.* The *Vat-1* allele from PI 161375 is amplified by the specific marker Z1431 and confers resistance to CMV upon inoculation by C9, GWD2, NM1, and putatively by C4. We proposed to name *Vat-2* the additional locus present in PI 161375. This allele confers at least resistance to CMV upon inoculation by GWD. *Vat-2* is likely tightly linked to *Vat-1* in PI 161375 because it cosegregated with *Vat-1*during the breeding program for constructing Margot when aphid resistance was introgressed and selected at each generation using the NM1 clone. In addition, this locus remained evident in a quasi-isogenic line, (data not shown) obtained after fifteen back-crosses and selection for resistance using the NM1 clone. This quasi-isogenic line, resistant to CMV inoculated by NM1 and GWD shares 99.99 % of its genome with the susceptible recurrent parent, and therefore the 0.01 % remaining contained *Vat-1* and *Vat-2*. Védrantais, which served as the susceptible control, was surprisingly resistant to CMV inoculated by C4 (Table 1) and because Védrantais contains neither *Vat-1* nor *Vat-2* alleles detected in PI 161375, other(s) locus or allele(s) is (are) likely involved in resistance to CMV when inoculated by C4. In the same way, Smith Perfect, Canton and HSD2455 shared susceptibility to CMV when inoculated by NM1, therefore do not carry the *Vat-1* allele detected in PI 161375, and resistance to CMV when inoculated by C6, a resistance elicited neither by *Vat-1* nor by *Vat-2* detected in PI 161375.

A 1-Mb region that contains *Vat-1* exhibits the highest concentration of presence/absence gene variation polymorphisms found in the melon genome [27], and this type of polymorphism is often related to phenotypic diversity in resistance to pathogens. This region also exhibits the highest density of resistance genes in the melon genome [28]. Twenty-three genes of the NBS-LRR family have been identified in this 1-Mb region [29], that potentially corresponds to less than 20 cM, and these genes are candidates for *Vat-2*. An homolog of *Vat-1* in 90625 shared 93.8 % identity at the DNA level and 92.3 % at the protein level with *Vat-1* from PI 161375 [30]. The numerous duplications in the region have made accurate sequencing difficult, therefore comprehensive crossing between molecular and phenotypic data is required to fully understand the genetic control of resistance to aphid and resistance to virus triggered by aphids. The use of transgenic lines will clearly help to decipher the role of each locus in this cluster.

### What have we learned from the pleiotropic resistance mediated by the *Vat* gene?

Recently, plant/aphid interactions have been included in the general framework of the plant immune system developed for plant/pathogen interactions [31]. Given that the *Vat-1* gene encodes an NBS-LRR protein that is similar to numerous resistance genes to pathogens, resistance is likely initiated by the specific recognition of aphid effector proteins [22]. Recognition activates signaling cascades; the only NBS-LRR gene-controlled cascade identified among plant resistance to aphids is the salicylic acid signaling pathway activated by *Macrosiphum euphorbiae* in *Mi-1*-tomato plants [32, 33]. This pathway also elicits resistance to virus [34]. In *Vat*-melon, the cascade elicits plant defenses against aphids and viruses. Physiological responses at *A. gossypii* feeding sites include very early deposits of callose and lignin in the cell walls, an increased peroxidase activity, phenol synthesis and a micro-oxidative burst [35, 36]. These physiological responses constitute a microscopic hypersensitive response in the leaf tissues of *Vat* plants infested by *A. gossypii*. Some miRNAs that regulate gene expression at a post-transcriptional level have been shown to be up-regulated during the early stages of aphid infestation in *Vat*-resistant plants and down-regulated in susceptible plants [37].

**Fig. 4:**
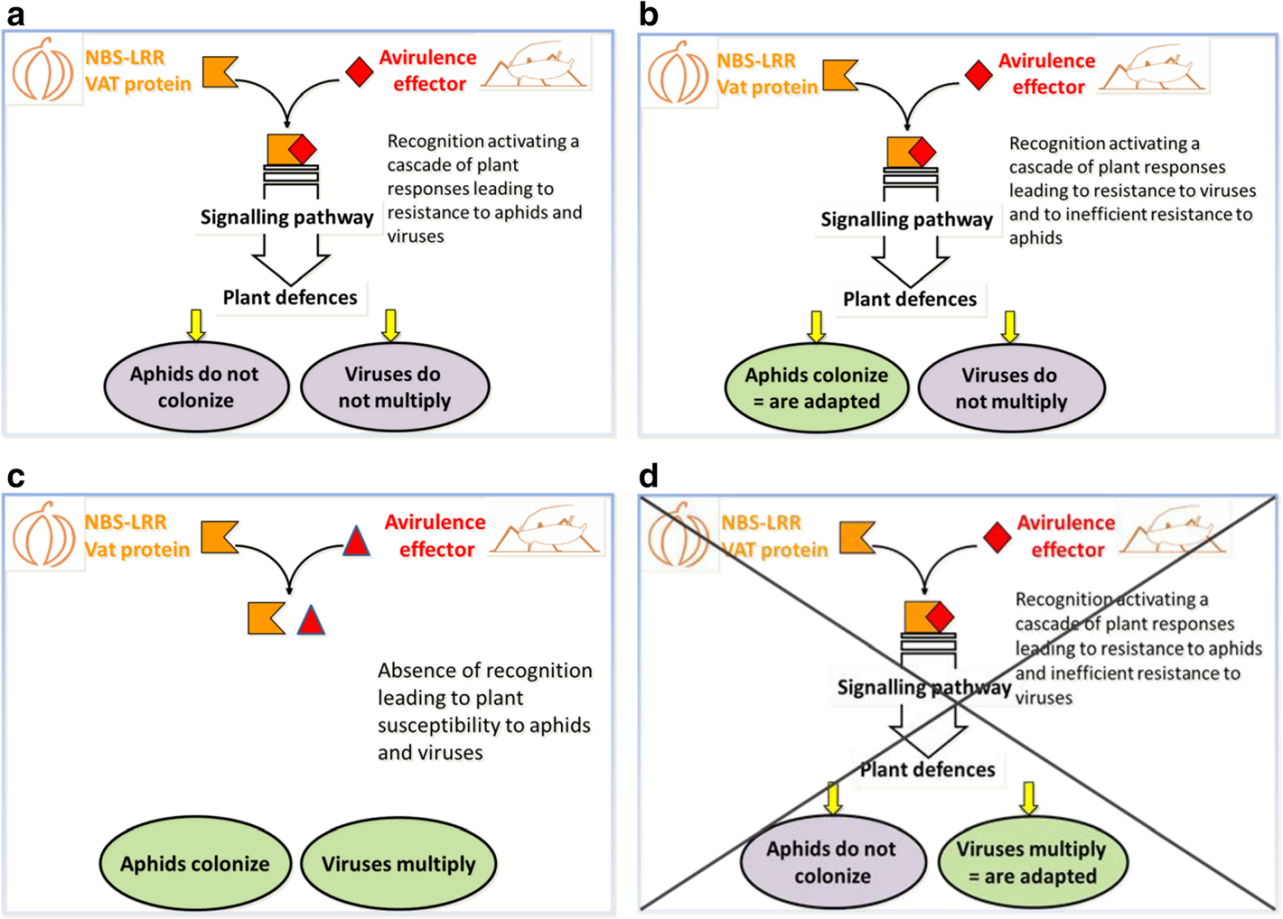
Model for *A. gossypii*/*Vat*-melon plant interaction: the 3 cases observed, **a** resistance to aphids and viruses, **b** susceptibility to aphids and resistance to viruses, **c** susceptibility to aphids and viruses, **d** resistance to aphids and susceptibility to viruses, which was not observed

Some phenotypes observed in the *Vat* transgenic line are consistent with the framework described above. Resistance is initiated by the specific recognition of an aphid effector that activates signaling cascades that elicit plant defenses against aphids and viruses (Fig. 4a). Phenotypes observed with the NM1 and C9 clones matched this scheme: both clones trigger resistance to CMV and were unable to fully colonize the *Vat* transgenic line. Considering resistance to virus triggered by aphids and low acceptance, 52 interactions among 117 studied in the natural diversity also matches this scheme. When there was no recognition of the aphid effector, plant defenses against aphids and viruses were not elicited (Fig. 4c). The phenotypes observed with the C6 and GWD clones matched this scheme given that these clones did not mediate resistance to CMV and fully colonized the *Vat* transgenic line. Considering resistance to virus triggered by aphids and low acceptance, 36 interactions among 117 studied in the natural diversity also matches this scheme. No aphid effector specifically recognized by an NBS-LRR resistance protein has been described to date; however, dozens of avirulence genes have been identified in plant pathogens, such as bacteria, fungi and oomycetes. These avirulence genes seem to be subject to high-speed diversifying selection [38], and this phenomenon might also be true for avirulence genes in aphids.

A third group of phenotypes in the *Vat*-transgenic line did not fit the framework. The GWD2 clone triggered resistance to CMV, therefore the specific recognition of the GWD2 effector that activates the signaling cascades that elicit plant defenses must have occurred, but GWD2 colonized the *Vat* transgenic line. We hypothesized that the decoupling of resistance to aphid from resistance to virus was due to aphid adaptation, thus allowing aphids to colonize plants even when plant defenses were elicited (Fig. 4b). The clone CUC1 partially fits this scheme. This clone fully colonized the transgenic line but appeared to occasionally mediate resistance to CMV (few plants did not exhibit symptoms). CUC1 might produce a low quantity of its avirulence effector, and the cells (and therefore the plant) receiving the virus particles without the effector allowed the virus to spread systemically. However, plants with all cells receiving both virus particles and the effector did not permit viral multiplication.

In the natural diversity, the phenotype ‘resistance to virus triggered by aphids’/‘susceptibility to aphids’ was frequent. Of the 74 cases of resistance to CMV triggered by aphid clones, we observed 26 cases with acceptance scores ≥5 and still more cases, 47, with colonization scores ≥6. The C6, CUC6, and C4 clones colonized all melon accessions in which they triggered resistance to CMV. The CUC1 and GWD2 clones colonized all melon accessions but one in which they triggered resistance to CMV. ‘Colonization’ that results from acceptance, daily fecundity, pre-reproductive period and clone mortality exhibits quantitative variation among clones sharing the same MLG and is hypothesized to be controlled by several aphid genes [20]. Adaptation to plant resistance could result from polymorphisms and/or regulation of these genes. Comparative analyses of aphids feeding on *Vat* and non-*Vat* plants has revealed that miRNAs are differentially regulated during resistant and susceptible interactions [39]. The abundance of Piwi-interacting RNA-like sequences (originating from repeat elements in the genome) in aphids feeding on *Vat* plants raises questions about their involvement in aphid responses to *Vat*-mediated resistance. In the Russian wheat aphid (*D. noxia*), differences in the DNA methylation level of four genes that presumably encode proteins and enzymes in aphid salivary glands, in addition to high levels of polymorphisms, were noted between two clones exhibiting different virulences on host plants [40].

### Are viruses able to adapt to *Vat* resistance triggered by aphids?

A fourth putative scheme might occur (Fig. 4d), *i.e.* virus adaptation to defenses triggered by *A. gossypii* probing on *Vat* plants. The expected phenotype is ‘susceptibility to virus when inoculated by an aphid clone incapable of colonizing *Vat* plants’. This double phenotype was never observed in the transgenic line, suggesting viral adaptation did not occur. Nevertheless, this double phenotype was observed twice in natural melons; both instances involved Anso 77 and the clones C9 and CUC1. Does this mean that both clones triggered resistance in Anso but that the CMV-I17F isolate had adapted to this resistance? This is an unlikely explanation given that Anso 77 was highly resistant to CMV when resistance was triggered by NM1, C4, GWD2, GWD, or CUCU3. Resistance to colonization of Anso 77 by C9 and CUC1 was most likely conferred by gene(s) other than the *Vat* gene; this other gene exclusively acts against aphids, similar to all other resistance genes described in crops. The inability of viruses to adapt to defenses triggered by the puncturing of *Vat* plants by *A. gossypii* was confirmed by experimental evolution biotests wherein CMV, as well as ZYMV, failed to evolve when facing plant defenses triggered by aphid probing. Therefore, regarding the virus, *Vat*-mediated resistance to viruses appeared durable in contrast to several NBS-LRR resistance to virus, such as *Tm-2* or *Sw-5.* The practical use of these genes is limited because the resistance conferred by the genes can be overcome by naturally occurring strains [41, 42]. While common NBS-LRR resistances to virus are triggered by an Avr viral protein [43], the absence of viral involvement in the recognition of the *Vat* protein was clearly established by the fact that *Vat* plants are systemically infected when viruses are mechanically transmitted or transmitted by *M. persicae* [25]. This phenomenon could ensure *Vat* durability against non-persistently transmitted virus. To overcome *Vat*-mediated resistance, viruses should evolve toward faster cell-to-cell movements after *A. gossypii* inoculative puncturing, to escape the resistance mechanisms induced by an *A. gossypii* effector. Such evolution has not been observed in our experimental evolution biotests.

## Conclusion

The resistance to viruses that is conferred on melon by *A. gossypii* puncturing appears durable, and this resistance is controlled by at least two loci, *Vat-1* and *Vat-2*, tightly linked. Different alleles likely mediate resistance to virus upon inoculation by specific aphid clones. Unfortunately, because numerous aphid species transmit viruses to melon crops, *Vat* resistance does not always significantly reduce viral epidemics in melon fields [44].

Resistance to *A. gossypii* in melon plants appeared very strong for only one clone, NM1, and partial or null for other clones. Building complex resistance to aphids required a better understanding of the genetic control using an aphid biotype-base strategy.

Considering ‘Acceptance’ and ‘Resistance to virus elicited by aphids’, 97 % of interactions in natural diversity fit with three schemes we proposed. These schemes suggested that aphid clones were adapted to plant resistance because their avirulence factors did not trigger resistance or because they could colonize the plants even if they elicited the defenses. If the latter is a general mechanism of plant resistance/aphid interactions, it would make the identification of avirulence factors challenging, given that adapted and non adapted clones could share a same avirulence effector interacting (directly or indirectly) with the protein encoded by the resistance gene.

## Methods

### Aphid clones

*A. gossypii* clones infesting melon belong to an host race group specialized on Cucurbitaceous [12]. The nine clones used in this study were collected in France (C4, C9, CUC1, CUC6, CUCU3, NM1) or in the lesser Antilles (C6, GWD, GWD2) on Cucurbits: C4 on *Ecballium elaterium*, NM1 on *Cucurbita* maxima, and all other clones on melon. NM1 was collected in 1978 by Labonne G. and used for the initial description of *Vat* [24, 25], it has since been maintained in our lab as a reference clone. Other clones were collected either on the experimental sites of INRA (PACA and Antilles-Guyane), or on the experimental sites of CEFEL (Centre d’Expérimentation des Fruits et Légumes) and De Ruiter seed company with their permission. The nine clones were characterized using DNA amplification at 8 microsatellite loci specific to the *A. gossypii* genome [12, 45]. The allele size at each locus was identified by comparison with a molecular size standard using the software GeneMapper v3.7 (Applied Biosystems, Foster City, California, USA), and a multilocus genotype (MLG) was then assigned to each aphid clone (Additional file 3). For simplification, clones are named according to their MLGs throughout the study.

Clones were maintained by synchronous mass rearing on melon Védrantais at 24 °C:18 °C under a 16 h:8 h photoperiod. Five- to seven-day-old aphids were used to infest plantlets at the two-leaf stage for biotests conducted in the same climatic conditions.

### Virus material

Three virus species were used, all transmitted in a non-persistent manner by aphids. The CMV is the type member of the plant virus genus Cucumovirus. We used the isolate I17F, belonging to the IA group (accession numbers HE793683, HE793684, Y18137), collected in France in 1975 on tomato. ZYMV and WMV are both belonging to the potyvirus genus. We used the isolate ZYMV-E15 (accession numbers JN861005 and AY189003) collected in France in 1979 on melon and the isolate WMV-FMF00-LL2 (accession number EU660578) collected in France in 2000 on zucchini. These virus isolates are reference isolates for France and were collected and maintained according to the national regulations.

### Plant material

Two *Vat* transgenic lines (TR3 and TR4) and thirteen melon cultivars or accessions were used in the biological tests. TR3 and TR4 were obtained from two independent events of *Agrobacterium*-mediated transformation on Védrantais melon by the insertion of an 11-kb genomic DNA sequence that included the *Vat* allele under the control of its own promoter isolated from PI 161375 [21].

Melon accessions or lines have diverse geographical origins: Védrantais, Margot and Anso 77 are from Europe; 90625, PI 161375, PI 164723, AM51, Canton and San Ildefonso are from Asia; PI 482398, PI 224770 and HSD 2455 are from Africa, and Smith Perfect is from America. Some of these lines were chosen for their resistance to *A. gossypii* [20, 46]. In particular, Margot is a Charentais cultivar in which resistance to *A. gossypii* was introgressed from PI 161375, Kanro Makuwa and Ginsen Makuwa. PI 482398, Margot, AM51, PI 161375 and San Ildefonso amplified the specific marker developed from the *Vat* gene, Z1431 described in the Additional file 4, other accessions did not.

All seeds were supplied by the Vegetables Genetic Ressources Center of UR 1052 – INRA. Plantlets were grown in an insect-proof greenhouse until they developed one or two leaves and were subsequently used for biological tests.

As a preliminary experiment, we checked the susceptibility of all accessions to viruses mechanically inoculated as described in [47]. Ten plantlets of all accessions or lines were inoculated with CMV, and 10 plantlets of Margot and Védrantais were also inoculated with ZYMV and WMW. All plantlets exhibited mosaic symptoms 7–10 days after inoculation and therefore were susceptible to the virus tested.

### Assessment of resistance to aphid and virus in melon lines and accessions

Two types of tests were conducted from 2004 to 2015 using the nine aphid clones on the different melon lines and accessions. The first test characterized resistance to CMV when inoculated by an *A. gossypii* clone, whereas the second test characterized plant acceptance of an *A. gossypii* clone and the clone’s ability to colonize the plant.

#### Assessment of the resistance to virus when inoculated by an A. gossypii clone

Aphids from mass rearing were transferred to CMV (isolate I17F)-infected leaves of Védrantais melon plants for 10 min virus acquisition. Batches of 10 aphids were subsequently deposited on plantlets from the different accessions for virus inoculation. After 15 min, the aphids were removed. The plants were sprayed with pyrimicarb (NM1 clone) or endosulfan (all others clones) and placed into an insect proof glasshouse. The number of infected plants was determined 20 days after inoculation by visual assessment of symptoms. Each test was conducted with one aphid clone on a sub-set of accessions. At least 9 plantlets of each accession and 20 plantlets of the *Vat* transgenic lines were tested. For aphid clone/melon combinations exhibiting intermediate percentage of infected plants, new tests were performed to obtain an accurate interval of confidence, all-in 50 tests were performed. Number of plantlets observed for each combination was given in the Additional file 5. To compare all combinations (melon accession/aphid clone), we followed the procedure proposed by [20]. Two reference lines, Védrantais (susceptible) and Margot (known for carrying resistance to CMV inoculated by NM1 and C9 clones) were included in all of the tests, and the data obtained were pooled and used to define the references. The reference line/aphid clone responses were compared using a Monte Carlo exact test with a χ^2^ statistic and, considering the number of comparisons performed, α = 0.0003. ‘Plant response to CMV’ scores were given to the each reference combination. Afterwards, the plant response to CMV triggered by each aphid clone on each melon accession was compared with the plant response to CMV observed on the two reference lines in the same test and was scored as Margot, Védrantais or intermediate.

#### Assessment of the resistance to A. gossypii clones

To assess the aphid acceptance and ability to colonize melon plants, 10 adult aphids were deposited on plantlets. Three days later, the number of aphids remaining on the plantlets was recorded as the ‘Acceptance’ parameter. Seven days after aphid deposition, the adults were counted, and the density of nymphs was estimated on a scale of 0 to 6. The ‘Colonization’ parameter at 7 days was calculated as [density of nymphs + ln(number of adults + 0.001)]. The ‘Acceptance’ and ‘Colonization’ parameters were collected for at least 8 plantlets of each melon accession and 20 of the *Vat* transgenic TR3. Each test was conducted with one aphid clone on a sub-set of melon accessions. When the accuracy of ‘Acceptance’ or ‘Colonization’ parameters was not satisfying for a combination aphid clone/melon accession, the combination was tested again to obtain an accurate interval of confidence. All-in 46 tests were performed. Number of plantlets observed for each combination was given in the Additional file 5. To compare all combinations (melon accession/aphid clone), we followed the same procedure outlined for ‘Plant response to CMV’, *i.e.* Védrantais and Margot were included in all tests and used as references. Because the ‘Acceptance’ and ‘Colonization’ parameters are quantitative, the procedure was based on a non-parametric analysis of the data (Steel-Dwass-Critchlow-Fligner analysis with Bonferroni correction).

All statistical analyses were conducted with XLSTAT software (AddinSoft, Paris, France).

### Assessment of virus ability to adapt to *Vat*-mediated resistance

To assess the virus ability to adapt to *Vat*-mediated resistance we conducted sequential virus transmissions on *Vat*-carrying Margot plants with CMV, ZYMV and WMV with NM1 *A. gossypii* clone. Initial inoculations were conducted following the procedure described above for plant rearing and virus sources with the exception of ZYMV, which used zucchini squash plants (cv Diamant) as the virus source. NM1 aphids collected in mass rearings were starved for 2 h and then transferred to CMV-infected leaves of Védrantais melon for 3 min virus acquisition. First, batches of 10 aphids were transferred to Margot plants for a 2-hour inoculation period. In the following tests we doubled the number of vectors or used the C9 clone as vector. When infected Margot plants were obtained, they were used to conduct the sequential Margot to Margot transmission experiments. Védrantais plants were included as controls in each test.

## Availability of supporting data

The data sets supporting the results of this article are included within the article and its additional files.

## Additional files

Additional file 1:
**Data set of resistance tests for melon accessions and lines.** (XLSX 1385 kb) Additional file 2:
**Checking for C4 clone capacity to transmit CMV on a set of Cucurbits.** Percentage of plants with CMV symptoms after inoculation by five *A. gossypii* belonging C4 clone. n = number of plants inoculated, Symptoms were observed 3 weeks after inoculation (DOCX 14 kb) Additional file 3:
**Characteristics of the nine** ***Aphis gossypii*** **clones.** Multilocus genotypes (MLGs) identified as different allelic combinations at the eight microsatellite loci (called Ago) in clones used in this study. The size of each allele was indicated in base pairs. (DOCX 22 kb) Additional file 4:
**PCR-based marker specific of the** ***Vat*** **gene from [** 21 **].** (DOCX 21 kb) Additional file 5:
**Number of plants used to establish scores for Resistance to CMV and Acceptance/Colonization of 123 melon/aphid interactions.** (DOCX 26 kb)

## Abbreviations

CABYV: Cucurbit aphid-borne yellows virus
CMV: Cucumber mosaic virus
MLG: multilocus genotype
NBS-LRR: nucleotide-binding site (NBS)-leucine-rich repeat (LRR)
PRSV: Papaya ringspot virus
WMV: Watermelon mosaic virus
ZYMV: Zucchini yellow mosaic virus
