# Supplementary material for: NBS-LRR-mediated resistance triggered by aphids: viruses do not adapt; aphids adapt via different mechanisms
Source: BMC Plant Biol. 2016 Jan 22;16:25. doi: 10.1186/s12870-016-0708-5 (PMC4722753; doi:10.1186/s12870-016-0708-5)
Supplement: Additional file 2: — Checking for C4 clone capacity to transmit CMV on a set of Cucurbits. Percentage of plants with CMV symptoms after inoculation by five A. gossypii belonging C4 clone. n = number of plants inoculated, Symptoms were observed 3 weeks after inoculation (DOCX 14 kb) [file 12870_2016_708_MOESM2_ESM.docx]

**Additional file 2 Checking for C4 capacity to transmit CMV on a set of Cucurbits.**

Percentage of plants with CMV symptoms after inoculation by five *A. gossypii* belonging C4 clone. n = number of plants inoculated, Symptoms were observed 3 weeks after inoculation

|  | n | % CMV-infected plants |
| --- | --- | --- |
| Zucchini squash | 20 | 60 % |
| Cucumber | 20 | 60 % |
| Melon WMR 29 | 20 | 70 % |
| Melon Ouzbèque 1 | 20 | 65 % |
| Melon Védrantais | 20 | 0 % |
