# Supplementary material for: NBS-LRR-mediated resistance triggered by aphids: viruses do not adapt; aphids adapt via different mechanisms
Source: BMC Plant Biol. 2016 Jan 22;16:25. doi: 10.1186/s12870-016-0708-5 (PMC4722753; doi:10.1186/s12870-016-0708-5)
Supplement: Additional file 3: — Characteristics of the nine Aphis gossypii clones. Multilocus genotypes (MLGs) identified as different allelic combinations at the eight microsatellite loci (called Ago) in clones used in this study. The size of each allele was indicated in base pairs. (DOCX 22 kb) [file 12870_2016_708_MOESM3_ESM.docx]

**Additional file 3 Characteristics of the nine *Aphis gossypii* clones**. Multilocus genotypes (MLGs) identified as different allelic combinations at the eight microsatellite loci (called Ago) in clones used in this study. The size of each allele was indicated in base pairs.

| Collect characteristics of aphid clones | | | | | | Microsatellite loci | | | | | | | |  |
| --- | --- | --- | --- | --- | --- | --- | --- | --- | --- | --- | --- | --- | --- | --- |
| Name | Year | Area | Host plant | MLG | Ago24 | | Ago53 | Ago59 | Ago66 | Ago69 | Ago84 | Ago89 | Ago126 | |
| Ecballium | 2012 | France | *E. elaterium* | **C4** | 153-153 | | 116-116 | 180-182 | 152-152 | 109-114 | 112-116 | 150-150 | 176-176 | |
| Gwada A | 2011 | Antilles | *Vat-*melon | **C6** | 153-153 | | 116-116 | 180-182 | 152-152 | 109-114 | 112-118 | 150-150 | 176-176 | |
| 4-104 | 2004 | France | *Vat*-melon | **C9** | 153-157 | | 116-116 | 182-182 | 152-152 | 109-114 | 112-118 | 150-150 | 176-176 | |
| PI 224770 | 2009 | France | melon | **CUC1** | 153-157 | | 116-116 | 182-207 | 147-152 | 109-114 | 112-124 | 150-150 | 176-176 | |
| DR B | 2009 | France | *Vat*-melon | **CUC6** | 153-157 | | 116-116 | 182-182 | 147-152 | 109-114 | 112-124 | 150-150 | 176-176 | |
| CEFEL | 2009 | France | melon | **CUCU3** | 157-157 | | 110-116 | 147-147 | 147-152 | 109-116 | 118-118 | 150-152 | 176-176 | |
| Gwada | 2011 | Antilles | *Vat*-melon | **GWD** | 157-157 | | 113-116 | 163-182 | 152-152 | 109-115 | 124-124 | 150-150 | 176-176 | |
| Gwada E | 2011 | Antilles | *Vat*-melon | **GWD2** | 157-157 | | 116-116 | 163-182 | 152-152 | 109-115 | 124-124 | 150-150 | 176-176 | |
| NM1-lab | 1978 | France | *Cucurbita* sp. | **NM1** | 153-153 | | 113-116 | 184-217 | 152-156 | 109-115 | 116-116 | 150-158 | 176-177 | |
