# Supplementary material for: NBS-LRR-mediated resistance triggered by aphids: viruses do not adapt; aphids adapt via different mechanisms
Source: BMC Plant Biol. 2016 Jan 22;16:25. doi: 10.1186/s12870-016-0708-5 (PMC4722753; doi:10.1186/s12870-016-0708-5)
Supplement: Additional file 4: — PCR-based marker specific of the Vat gene from [ 21 ]. (DOCX 21 kb) [file 12870_2016_708_MOESM4_ESM.docx]

**Additional file 4 PCR-based marker specific of the *Vat* gene** from ([Dogimont et al., 2014](#_ENREF_14))

| Name | Primer sequence (5’-3’) | Size (bp) | Localization |
| --- | --- | --- | --- |
| Z1431 | F: ATGCAAAGAGTTTGAAGATG  R:GAAGATTACATAACATCAACGA | 858 bp | Exon 2 |

PCR conditions: 4 min at 94°C; (30 sec at 94°C; 30 sec at 57°C; 1 min at 72°C) during 35 cycles; 10 min at 72°C
