# Supplementary material for: NBS-LRR-mediated resistance triggered by aphids: viruses do not adapt; aphids adapt via different mechanisms
Source: BMC Plant Biol. 2016 Jan 22;16:25. doi: 10.1186/s12870-016-0708-5 (PMC4722753; doi:10.1186/s12870-016-0708-5)
Supplement: Additional file 5: — Number of plants used to establish scores for Resistance to CMV and Acceptance/Colonization of 123 melon/aphid interactions. (DOCX 26 kb) [file 12870_2016_708_MOESM5_ESM.docx]

**Additional file 5 Number of plants used to establish scores for Resistance to CMV and Acceptance/Colonization of 123 melon/aphid interactions.**

|  | Resistance to CMV | | | | | | | | | |  | | Acceptance/Colonization | | | | | | | | |
| --- | --- | --- | --- | --- | --- | --- | --- | --- | --- | --- | --- | --- | --- | --- | --- | --- | --- | --- | --- | --- | --- |
|  | C6 | C9 | CUC1 | CUC6 | CUCU3 | GWD | GWD2 | NM1 | C4 |  | | C6 | | C9 | CUC1 | CUC6 | CUCU3 | GWD | GWD2 | NM1 | C4 |
| TR3 | 20 | 20 | 80 | nt | nt | 20 | 20 | 20 | nt |  | | 20 | | 29 | 40 | nt | nt | 20 | 20 | 30 | nt |
| TR4 | nt | nt | nt | nt | nt | 20 | nt | 20 | nt |  | | nt | | nt | nt | nt | nt | nt | nt | nt | nt |
| PI 482398 | 20 | 20 | 20 | 10 | 10 | 10 | 10 | 10 | 15 |  | | 14 | | 20 | 15 | 10 | 10 | 10 | 15 | 20 | 15 |
| Margot | 60 | 113 | 95 | 35 | 40 | 55 | 60 | 114 | 55 |  | | 30 | | 108 | 55 | 34 | 45 | 51 | 45 | 119 | 38 |
| AM51 | 20 | 20 | 20 | 10 | 10 | 10 | 20 | 10 | 14 |  | | 15 | | 20 | 15 | 25 | 25 | 25 | 15 | 10 | 14 |
| PI 161375 | 37 | 50 | 55 | 10 | 15 | 34 | 39 | 20 | 15 |  | | 25 | | 23 | 25 | 12 | 14 | 24 | 25 | 37 | 10 |
| San Ildefonso | 18 | 10 | 20 | 10 | 10 | 10 | 18 | 10 | 11 |  | | 10 | | 20 | 15 | 10 | 10 | 9 | 8 | 10 | 10 |
| Smith Perfect | 20 | 15 | 20 | 10 | 10 | 10 | 20 | 19 | 15 |  | | 15 | | 15 | 15 | 10 | 10 | 13 | 15 | 23 | 14 |
| Canton | 20 | 25 | 10 | 10 | 10 | 10 | 20 | 10 | 14 |  | | 15 | | 20 | 14 | 10 | 10 | 10 | 15 | 20 | 10 |
| HSD2455 | 18 | 10 | 19 | 9 | 10 | 10 | 17 | 15 | 15 |  | | 8 | | 20 | 10 | 10 | 10 | 10 | 14 | 10 | 15 |
| Anso 77 | 20 | 15 | 10 | 10 | 9 | 10 | 10 | 25 | 10 |  | | 14 | | 25 | 14 | 10 | 10 | 13 | 15 | 30 | 11 |
| PI 224770 | 20 | 40 | 10 | 20 | 25 | 30 | 20 | 20 | 15 |  | | 15 | | 9 | 20 | 10 | 10 | 13 | 20 | 6 | 15 |
| 90625 | 20 | 15 | 10 | 10 | 10 | 10 | 20 | 30 | 12 |  | | 14 | | 30 | 14 | 25 | 25 | 28 | 15 | 30 | 12 |
| PI 164723 | 58 | 115 | 95 | 35 | 39 | 55 | 60 | 120 | 54 |  | | 14 | | 19 | 14 | 10 | 10 | 13 | 15 | 8 | 14 |
| Védrantais |  | 20 | 20 | 80 |  |  | 20 | 20 |  |  | | 30 | | 110 | 55 | 35 | 45 | 51 | 45 | 122 | 34 |

*nt untested.
